# MicroRNA-218 inhibits tumor angiogenesis of human renal cell carcinoma by targeting GAB2

**DOI:** 10.3892/or.2022.8406

**Published:** 2022-09-13

**Authors:** Lijun Mu, Bing Guan, Juanhua Tian, Xiang Li, Qingzhi Long, Meiyu Wang, Wen Wang, Junjun She, Xudong Li, Dapeng Wu, Yuefeng Du

Oncol Rep 44: 1961–1970, 2020; DOI: 10.3892/or.2020.7759

Subsequently to the publication of the above article, the authors have realized that the cell migration and tube formation assay data portrayed in Figs. 2 and 4 in their paper were published with some inadvertent errors. Specifically, the photograph selected for the ACHN-SFM group was accidentally misused for the 769P/LV-miR-218 group in Fig. 2F. Secondly, the photograph for the 786O/LV-NC group in Fig. 2F was accidentally misused as the image for the 786O/shNC group in Fig. 4E; and thirdly, the photograph selected for the ACHN/shNC group in Fig. 4C was inadvertently misused for the ACHN/shNC group in Fig. 4D. These errors arose inadvertently as a consequence of the authors' mishandling their data; however, the authors were able to retrieve their original data, and have been able to reassemble these figures to show the data as was originally intended.

The revised versions of Figs. 2 and 4, featuring the corrected data panels for the 769P/LV-miR-218 group in Fig. 2F, the ACHN/shNC group in Fig. 4D and the 786O/shNC group in Fig. 4E, are shown on the next two pages. The revised data shown for these Figures do not affect the overall conclusions reported in the paper. All the authors agree with the publication of this corrigendum. The authors are grateful to the Editor of *Oncology Reports* for allowing them the opportunity to publish this corrigendum, and apologize to the readership for any inconvenience caused.

## Figures and Tables

**Figure 2.**
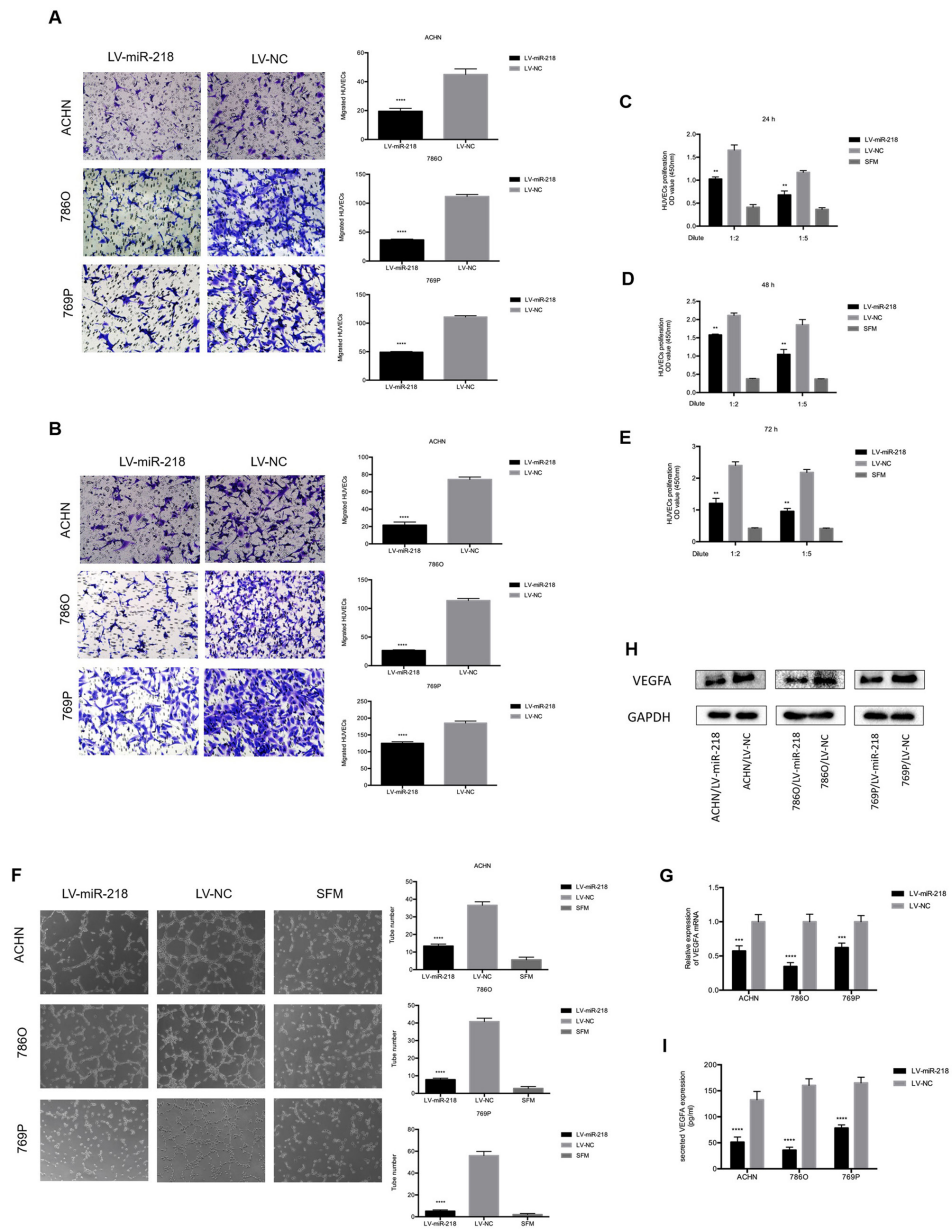
miR-218 inhibits HUVEC migration, proliferation and tube formation in vitro, and inhibits VEGFA expression. (A) Overexpression of miR-218 suppresses the recruitment of HUVECs in a co-cultured system. Cancer cells cultured in the bottom of a 24-well plate were used to recruit HUVEC seeding on the upper chamber of Transwell insets. ^****^P<0.0001 compared with the LV-NC group. (B) Overexpression of miR-218 decreased the recruitment of HUVECs through the conditioned medium (CM), which was collected from the ACHN, 786O and 769P/LV-miR-218 or LV-NC cells. HUVECs were seeded on the upper chamber of the Transwell insets within 16 h. The migrated cells in six random fields per well were counted (magnification, ×200). ^****^P<0.0001 compared with the LV-NC group. (C-E) miR-218 overexpression reduced the proliferation of HUVECs. HUVECs were treated with serum-free medium (SFM) or diluted CMs for 24, 48 and 72 h before the CCK-8 assay. **P<0.01 compared with the LV-NC group. (F) miR-218 overexpression suppressed the tube formation of HUVECs. HUVECs diluted in SFM or CMs were added into Matrigel-coated wells and incubated for 4 h. The representative images of tube-like structures were captured (left), and the tube number in the whole field was counted (magnification, ×100) (right). ^****^P<0.0001 compared with the LV-NC group. (G) Real-time PCR was used to analyze the expression level of VEGFA in ACHN, 786O and 769P cells transfected with LV-NC or LV-miR-218. ^***^P<0.001 and ^****^P<0.0001 compared to the LV-NC group. (H) The VEGFA protein level was analyzed by western blot analysis. (I) The concentration of secreted VEGFA protein in the CMs was determined by ELISA. ^****^P<0.0001 compared to the LV-NC group. These data are representative of three independent experiments. HUVECs, human umbilical vein endothelial cells; VEGFA, vascular endothelial growth factor A.

**Figure 4.**
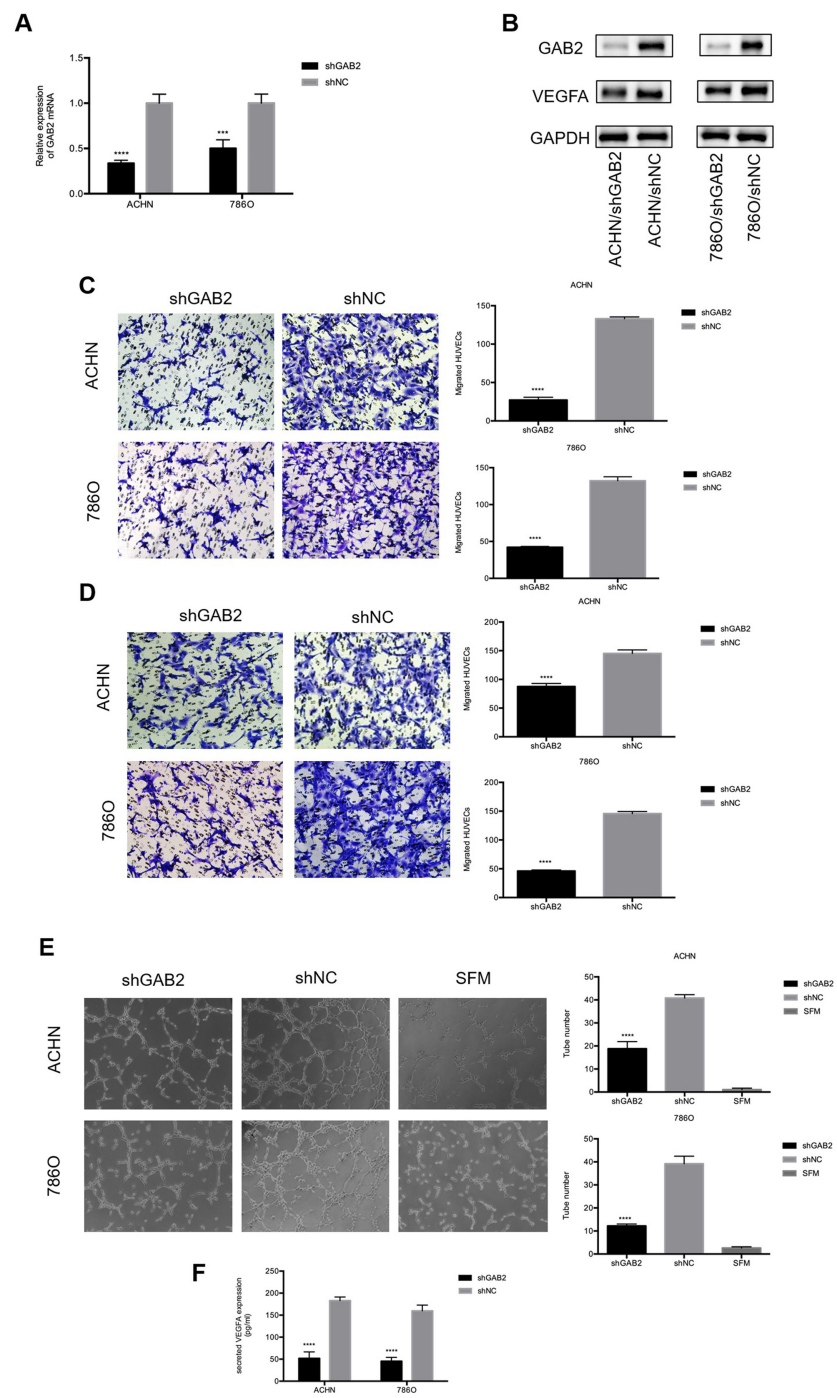
GAB2 plays a crucial role in RCC angiogenesis. (A and B) Real-time PCR and western blot analysis were used to confirm the knockdown of GAB2 in ACHN and 786O cells transfected with LV-shGAB2 both at the mRNA and protein level. GAB2 knockdown decreased VEGFA at the protein level. ^***^P<0.001 and ^****^P<0.0001 compared to the shNC group. (C) GAB2 knockdown decreased the recruitment of HUVECs in a co-cultured system. Cancer cells that were cultured in the bottom of a 24-well plate were used to recruit HUVECs. ^****^P<0.0001 compared to the shNC group. (D) GAB2 knockdown decreased the recruitment of HUVECs through the conditioned medium (CM) collected from the ACHN, 786O/LV-shGAB2, and ACHN, 786O/LV-shNC cells. The migrated cells in six random fields per well were counted (magnification, ×200). ^****^P<0.0001 compared to the shNC group. (E) GAB2 knockdown reduced the tube formation of HUVECs diluted in SFM or CMs. The representative images of tube-like structures are shown, and the tube numbers in the whole field were counted (magnification, ×100). ^****^P<0.0001 compared to the shNC group. (F) The concentration of secreted VEGFA protein in the CMs was determined by ELISA. The values were presented as the mean ± standard deviation (SD). GAB2, GRB2-associated binding protein 2; RCC, renal cell carcinoma; HUVECs, human umbilical vein endothelial cells; SFM, serum-free medium; CM, conditioned medium; VEGFA, vascular endothelial growth factor A.

